# Loss of Ahi1 Impairs Neurotransmitter Release and Causes Depressive Behaviors in Mice

**DOI:** 10.1371/journal.pone.0093640

**Published:** 2014-04-01

**Authors:** Liyan Ren, Xuanchen Qian, Lijing Zhai, Miao Sun, Zhigang Miao, Jizhen Li, Xingshun Xu

**Affiliations:** 1 Department of Neurology and Jiangsu Key Laboratory of Translational Research and Therapy for Neuro-Psycho-Diseases, the Second Affiliated Hospital of Soochow University, Suzhou City, Jiangsu Province, China; 2 The Institute for Fetology, the First Affiliated Hospital of Soochow University, Suzhou City, Jiangsu Province, China; 3 The Institute of Neuroscience, Soochow University, Suzhou City, Jiangsu Province, China; 4 Department of Neurology, Suzhou Kowloon Hospital, Suzhou City, Jiangsu Province, China; Emory University, United States of America

## Abstract

Major depression is becoming one of the most prevalent forms of psychiatric disorders. However, the mechanisms of major depression are still not well-understood. Most antidepressants are only effective in some patients and produce some serious side effects. Animal models of depression are therefore essential to unravel the mechanisms of depression and to develop novel therapeutic strategies. Our previous studies showed that Abelson helper integration site-1 (Ahi1) deficiency causes depression-like behaviors in mice. In this study, we characterized the biochemical and behavioral changes in Ahi1 knockout (KO) mice. In Ahi1 KO mice, neurotransmitters including serotonin and dopamine were significantly decreased in different brain regions. However, glutamate and GABA levels were not affected by Ahi1 deficiency. The antidepressant imipramine attenuated depressive behaviors and partially restored brain serotonin level in Ahi1 KO mice. Our findings suggest that Ahi1 KO mice can be used for studying the mechanisms of depression and screening therapeutic targets.

## Introduction

Depression is one of the most prevalent forms of psychiatric disorders and is a leading cause for mortality [1]. Lifetime prevalence of depression in the general population is 4.4–20% [2] and suicides occur in up to 15% of individuals with severe major depression [3]. Therefore, major depression is a serious public health problem and causes a considerable heavy psychological and economic burden for families [4]. To solve this severe public health problem, it is urgent to keep studying the pathophysiology of major depression and to develop new therapeutic targets and strategies. Many animal models of depression have been developed include environmental stress models, social stress models, pharmacological models and genetic models. Among all these models, genetic models have more stable phenotypes and are becoming a more common way to study the mechanisms of major depression.

Previous studies have demonstrated that the mutation of Abelson helper integrationsite-1 (*AHI1*) gene is one of the causes of Joubert syndrome, a neurodevelopmental disorder characterized by congenital malformation of the brainstem and agenesis or hypoplasia of the cerebellar vermis [5], [6]. The increasing line of evidences indicates that *AHI1* is associated with psychiatric diseases [7]–[10]. Our previous study demonstrated that conditional Ahi1 knockout (KO) in mice by a Cre-loxp system causes depressive behaviors [11], which are common symptoms in human psychiatric diseases. Neurochemical analysis indicated that Ahi1 deficiency impairs endocytic sorting of TrkB and increases degradation of TrkB in lysosome [11]. Since depression can be caused by multiple pathogenic pathways, it is important to investigate whether different mechanisms underlie the depressive phenotypes of Ahi1 KO mice. In this study, we found that Ahi1 deletion also impairs neurotransmitter release. Our findings suggest that Ahi1 KO mice can provide a genetic mouse model to study the pathogenesis of depression and to identify new therapeutic targets.

## Materials and Methods

### Ahi1 knockout (KO) mice

Ahi1^loxp/loxp^ mice were generated as described previously [11]. The floxed mice (Ahi1 loxp/loxp) were crossed with mice carrying an EIIa promoter-driven Cre transgene [(The Jackson Laboratory, B6.FVB-Tg (EIIa-Cre) C5379Lmgd/J)], which resulted in conditional Ahi1 knockout in the whole body. The resulting heterozygous mice were used to generate homozygous conditional KO (EIIa-Cre-Ahi1^−/−^) mice, in which Ahi1 was deleted in a wide range of tissues including germ cells. EIIa-Cre-Ahi1^−/−^ mice were viable and crossed with wild-type mice (C57BL/6J) to produce Ahi1 heterozygous (Ahi1^+/−^) mice without EIIa-Cre. Ahi1 heterozygous (Ahi1^+/−^) mice were used to generate Ahi1 KO (Ahi1^−/−^) mice. All animal procedures was approved by the University Committee on Animal Care of Soochow University and conducted in accordance with the guidelines of Animal Use and Care of the National Institutes of Health.

### Forced swimming test

Forced swimming test was performed as described previously [12]. Each mouse was placed in a glass cylinder (20 cm high, 15 cm in diameter) with water (23–25°C) to a depth 14 cm and was forced to swim for 6 min. Immobility time was recorded when the animal was making the minimum movements necessary to maintain floating in the water.

### Tail suspension test

The tail suspension test was performed according to the method outlined in previous reports with a minor modification [13], [14]. The method was based on the observation that a mouse suspended by the tail showed alternate periods of struggle and immobility. The distance between the tip of tail of each mouse and the desktop was about 35 cm. The mice were suspended for 6 min and the immobile time was recorded by using a stopwatch.

### Sucrose preference test

The sucrose preference test was performed as described previously with a minor modification [15]. Mice were housed individually and trained to drink water from two bottles for 24 h. In the next day, a bottle of water was replaced with a bottle of 1% sucrose solution. After 24 h, the positions of two bottles were exchanged. In the fourth day, mice were deprived water and food for 24 h. Before water and food were supplied, the bottles were weighted. After 24 h, the bottles were weighted again. The consumed amounts of 1% sucrose solution and water were calculated. The percentage of consumed sucrose to total drink was also calculated.

### Western blotting analysis

After mice were sacrificed, different brain region tissues including brainstem, cortex, amygdala, hippocampus, and hypothalamus were collected. Samples were solubilized in lysis buffer. After samples were centrifuged, supernatants were collected for Western blot analysis. The same amount of total proteins (about 50 μg) were loaded on a 12% sodium dodecyl sulfate-polyacrylamide gel electrophoresis (SDS-PAGE) and then electrotransferred to nitrocellulose membrane. Blots was blocked with 5% dry milk in phosphate-buffer saline/0.1%Tween 20 (PBST) for 2 h and then incubated with primary antibodies overnight with shaking at 4°C. In the next day, blots were washed with PBST and incubated with horseradish peroxidase-conjugated secondary antibody for 1 h at room temperature. After washing, blots were developed with the ECL chemiluminescence system (Thermo Company, West Chester, Pennsylvania, USA) and the immunoreactive bands captured on autoradiographic films (Kodak Company, Rochester, New York, USA). The densitometry of the bands was analyzed with Alpha Ease Image Analysis Software (Version 3.1.2).

### Determination the levels of monoamines and their metabolites

The test was performed as previous report with modification [16]. The amounts of monoamines and their metabolites were determined by using a high performance liquid chromatography (HPLC) method. After decapitation, each brain region was dissected on ice. Tissues were homogenized by an ultrasonic homogenizer following addition of 200 μl perchloric acid (0.4 M). The homogenates were placed on ice and then centrifuged at 10,000 g for 15 min. Perchloric acid (0.4 M) was added to 1 ml and injected into an HPLC system. The turnover of serotonin was assessed by the ratio of 5-HIAA/serotonin. Six mice in each group were used for the analysis of monoamine levels.

### Determination the levels of glutamate and GABA

The amount of glutamate and GABA were determined using an HPLC system as above. After adding 200 μl perchloric acid (0.4 M), we used 200 μl K_2_CO_3_ (2 M) to neutralize samples. Similarly, we added with 0.1 M K_2_CO_3_ to 1 ml to make sample derivatization and then injected into an HPLC system.

### Drug administration

Imipramine (20 mg/kg, Sigma-Aldrich Corp., St. Louis, USA) was dissolved in normal saline and freshly prepared before use. Mice were treated with imipramine or normal saline intraperitoneally (i.p.) for 3 weeks.

### Statistical analysis

All data were expressed as the mean ± SEM. Differences between two groups were determined with Student's t test. The differences among groups were compared with one-way analysis of variance followed by Tukey's multiple-comparison test. P<0.05 was considered statistically significant.

## Results

### Ahi1 KO caused depressive behaviors in young mice

**Figure 1:**
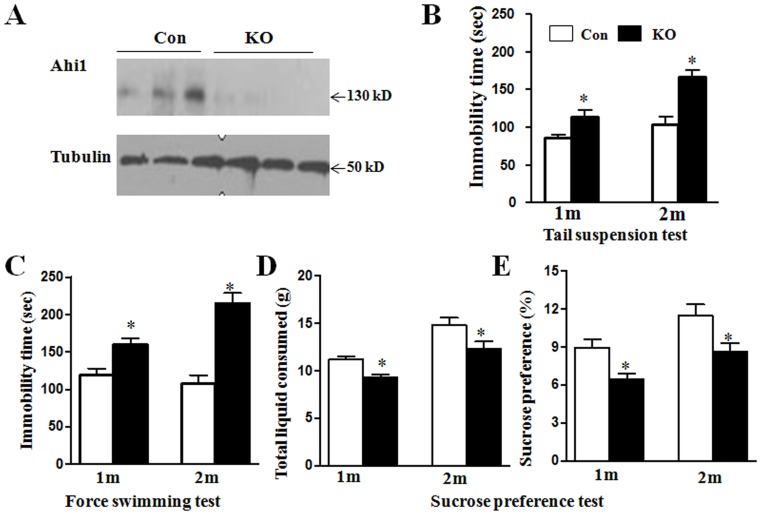
Ahi1 KO caused depressive behaviors in young mice. (A) Western blot analysis showed Ahi1 expression in the hypothalamic tissues from control mice and Ahi1 KO mice. (B) Immobility time in tail suspension test was shown in control mice and Ahi1 KO mice at the age of 1 m and 2 m. (C) Immobility time in forced swimming test was shown in control mice and Ahi1 KO mice at the age of 1 m and 2 m. (D and E) Sucrose preference test was performed and total consumed liquid (D) and the percentage of 1% sucrose (E) were calculated. *P<0.05 versus control mice. N = 6–8 mice.

Due to no differences in Ahi1 expression and behavioral tests between Ahi1 heterozygous mice (Ahi1^+/−^) and wild-type mice, we used Ahi1 heterozygous mice as controls to examine the phenotypes of Ahi1 KO (Ahi1^−/−^) mice. As shown in Fig. 1A there was almost no Ahi1 expression in Ahi1 KO mice. Although our previous study showed that Nestin-Cre conditional Ahi1 KO mice had depressive phenotype at the different ages [11], we further investigated whether Ahi1 KO mice have depressive phenotype. Mice at the age of 1 or 2 m were subjected to depressive behavioral tests. A significant decrease of immobility time was observed in Ahi1 KO mice for tail suspension test and forced swimming test as early as at 1 m (*P*<0.05, Fig. 1B and C). In sucrose preference test, total consumed liquid and the percentage of consumed sucrose water was markedly decreased in Ahi1 KO mice (*P*<0.05, Fig. 1D and E).

### Loss of Ahi1 affected depression-related neurotransmitter release with age

**Figure 2:**
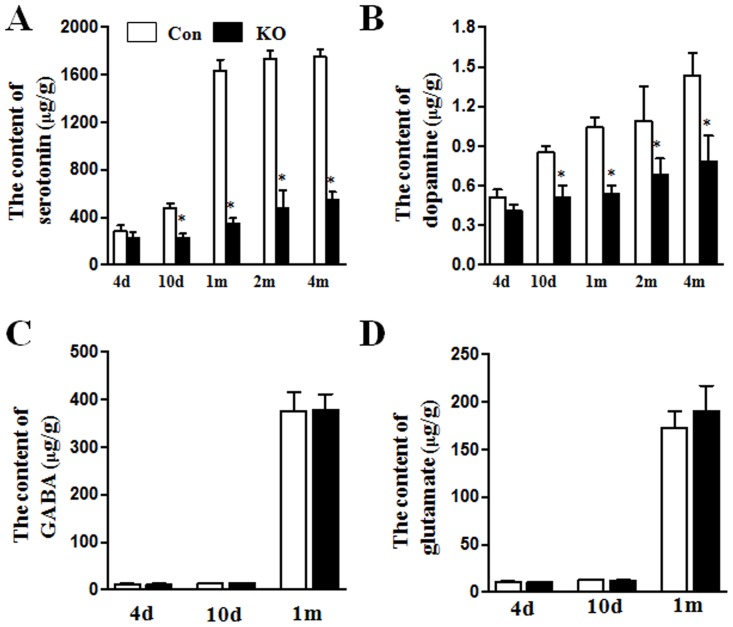
Loss of Ahi1 affected the increase of depression-related neurotransmitters with age. The levels of serotonin (A) and dopamine (B) in the brainstem tissues were examined at the age of 4 d, 10 d, 1 m, 2 m and 4 m. The content of GABA(C) and glutamate (D) in the region of brainstem was determined at the age of 4 d, 10 d, and 1 m. *P<0.05 versus control mice. N = 6.

To further understand the mechanisms of depressive behaviors by Ahi1 deficiency, we measured depression-related neurotransmitters in the brain to explicit whether low levels of neurotransmitters are related to the depressive behaviors of Ahi1 KO mice as in previous neurotransmitter hypothesis. Because brainstem is one of the most Ahi1 abundant brain regions [17], we measured the contents of neurotransmitters including serotonin, dopamine, glutamate, and GABA in brainstems by HPLC. At postnatal day 4, there was no difference for the content of serotonin in control and Ahi1 KO mice (*P*>0.05, Fig. 2A). However, after postnatal day 10, serotonin level was significantly increased in control mice, while only a slight increase of serotonin was observed in Ahi1 KO mice, demonstrating a markedly difference of serotonin level between control mice and Ahi1 KO mice (*P*<0.05, Fig. 2A). Similarly, dopamine level was not significantly elevated in Ahi1 KO mice as in control mice after postnatal day 10; therefore, Ahi1 mice showed much lower dopamine level compared with control mice (*P*<0.05, Fig. 2B). As a contrast, glutamate and GABA levels were not influenced in control and Ahi1 KO mice at 4 d-, 10 d-, and 1 m-old (*P*>0.05, Fig. 2C and D).

### Ahi1 deficiency decreased serotonin and dopamine levels in different brain regions

**Figure 3:**
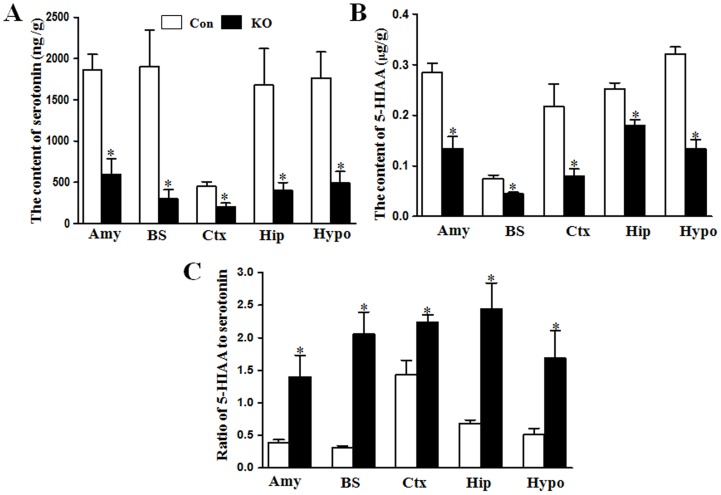
Serotonin and its metabolite 5-HIAA were decreased in different brain regions of Ahi1 KO mice. The levels of serotonin (A) and its metabolite 5-HIAA (B) were examined in amygdala (Amy), brainstem (BS), cortex (Ctx), hippocampus (Hip), and hypothalamus (Hypo) regions of control mice and AHi1 KO mice. (C) The ratios of 5-HIAA to serotonin in the five regions were calculated. *P<0.05 versus control mice. N = 6–8.

**Figure 4:**
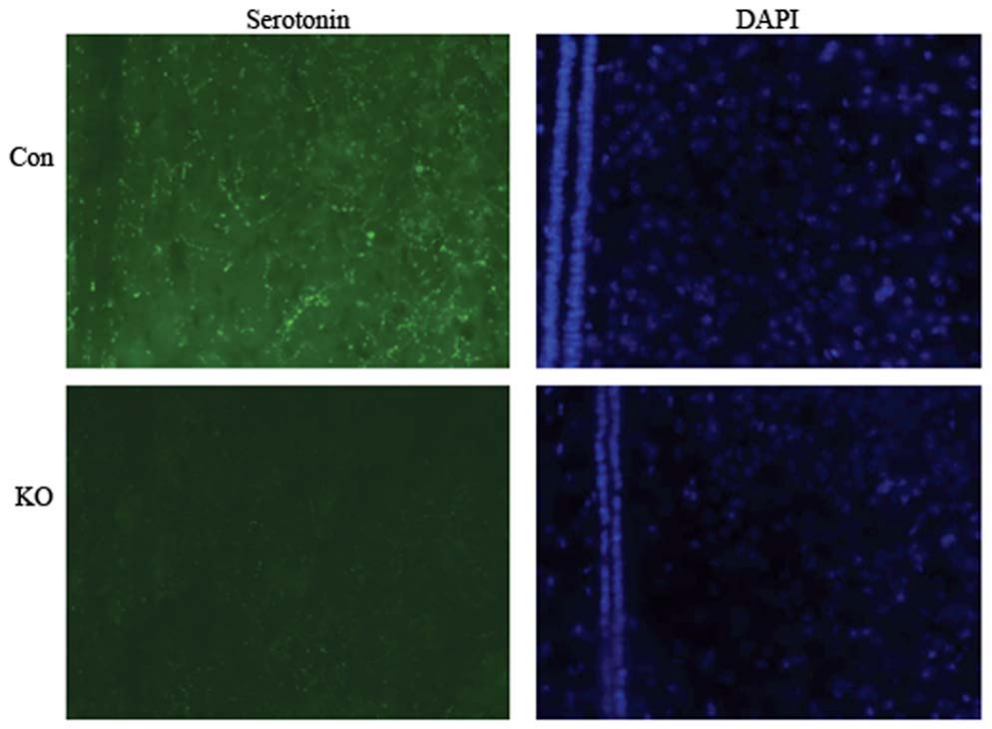
Fluorescent staining showed the decrease of serotonin in control and Ahi1 KO mice. The brain slices were stained with anti-serotonin antibody and DAPI. Significant decrease of fluorescent density of serotonin was observed in the hypothalamus area in Ahi1 KO mice (200X).

To confirm the decrease of depression-related neurotransmitters in different brain regions, we further measured serotonin levels in the cortex, hippocampus, hypothalamus, brainstem, and amygdale at 1-m-old mice. Although different brain regions had different levels of serotonin; serotonin level was significantly decreased in Ahi1 KO mice compared with control mice (*P*<0.05, Fig. 3A). 5-HIAA, a metabolite of serotonin, was also decreased in different brain regions in Ahi1 KO mice (*P*<0.05, Fig. 3B). However, the ratio of 5-HIAA to serotonin, namely serotonin turnover, was markedly elevated in Ahi1 KO mice (*P*<0.05, Fig. 3C), suggesting that reduced serotonin level may be due to excessive degradation, but not low production of serotonin. To demonstrate the decrease of serotonin in the brain, serotonin expression was further examined by immuno-fluorescent staining. As shown in Fig. 4, serotonin staining was much lower in the hypothalamus of Ahi1 KO mice than that of control mice, which was consistent with HPLC analytical results.

**Figure 5:**
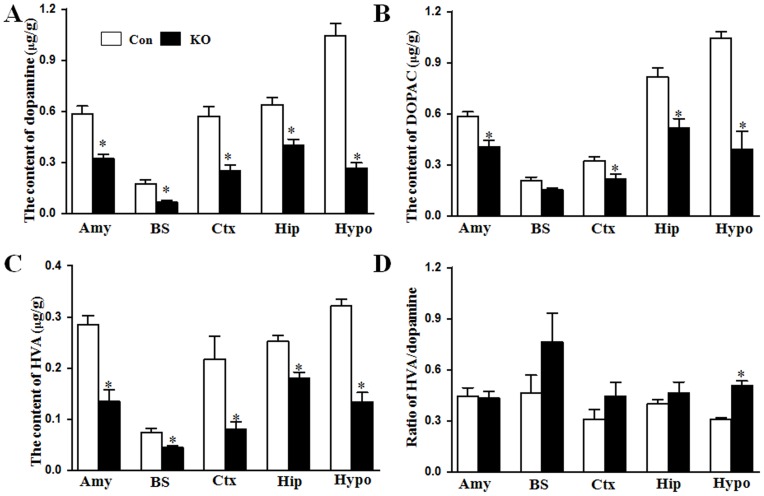
Ahi1 KO did not affect glutamate and GABA levels in mouse brains. The levels of dopamine (A) and its metabolite DOPAC (B) and HVA (C) were determined in different brain regions of control mice and AHi1 KO mice. (D) The ratios of HVA to dopamine in different brain regions were also calculated. *P<0.05 versus control mice. N = 6–8.

Dopamine level was also detected in 1 m-old control and Ahi1 KO mice. Like serotonin, dopamine level was decreased in different brain regions in Ahi1 KO mice (*P*<0.05, Fig. 5A). The metabolites of dopamine DOPAC and HVA were also decreased in Ahi1 KO mice (*P*<0.05, Fig. 5B and C). However, except hypothalamus, the ratios of HVA to dopamine were not changed in other brain regions (*P*>0.05, Fig. 5D).

### MAO activity was increased in Ahi1 KO mice

**Figure 6:**
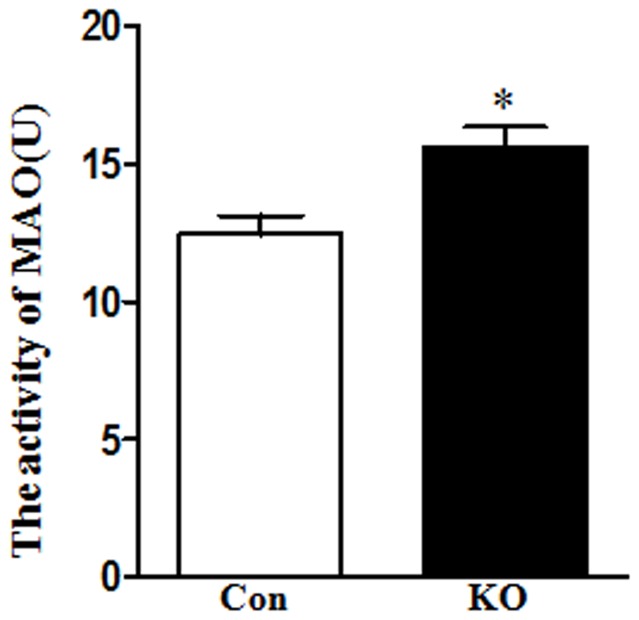
MAO activity was increased in brains of Ahi1 KO mice. MAO activity was determined by the ELISA method. MAO activity was significantly increased in Ahi1 KO mouse. *P<0.05, versus control mice. N = 6–8.

There are two isoforms of MAO enzymes in humans to catalyze the oxidative deamination of endogenous and exogenous monoamines: MAO-A and MAO-B. Serotonin, epinephrine and norepinephrine are mostly deaminated by MAO-A, but dopamine is degraded by both MAO-A and MAO-B. Therefore MAO activity is considered to be associated with psychiatric disorders and is therapeutic targets of depression and anxiety [18]. We measured the MAO activity in the brainstem of control and Ahi1 KO mice by an ELISA method. As shown in Fig. 6, MAO activity was significantly increased in Ahi1 KO mice compared with control mice (*P*<0.05).

### Impramine improved depressive behaviors and partially restored serotonin level in Ahi1 KO mice

**Figure 7:**
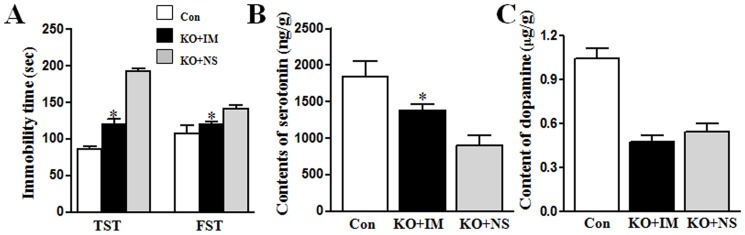
Impramine improved depressive behaviors and partially restored serotonin level in Ahi1 KO mice. (A) After mice were treated with antidepressants imipramine (20 mg/kg) for three weeks, the immobility time in tail suspension test (TST) and force swimming test (FST) was determined in control and Ahi1 KO mice. After treatment with imipramine (IM) or normal saline (NS), serotonin (B) and dopamine (C) levels in brains were determined. *P<0.05 versus normal saline-treated Ahi1 KO mice. N = 6–8.

Further, we examined whether depressive phenotype of Ahi1 KO mice is improved by imipramine, a clinical wildly used medication for major depression. Therefore, we treated Ahi1 KO mice with imipramine (20 mg/kg, i.p.) or saline for 3 weeks to determine the therapeutic effect of imipramine on the depressive phenotype. Three weeks later, immobility time in forced swimming test and tail suspension test was significantly decreased in imipramine-treated Ahi1 KO mice compared with saline-treated Ahi1 KO mice (*P*<0.05, Fig. 7A). With the improvement on the depressive phenotype, the serotonin level was also elevated in brains of imipramine-treated Ahi1 KO mice (*P*<0.05, Fig. 7B), however, dopamine level was not elevated (*P*>0.05, Fig. 7C).

## Discussion

In this study, we characterized the neurochemical changes of Ahi1 KO mice, especially on depression-related monoamine neurotransmitters such as serotonin and dopamine in different brain regions. These neurotransmitters did not increased in Ahi1 KO mice with age as in control mice, resulting in reduced serotonin and dopamine levels in adult KO mice (Fig. 2–5). At the same time, MAO activity was found to be increased in Ahi1 KO brains (Fig. 6). Finally, chronic imipramine treatment improved depressive phenotype and partially restored serotonin level in brains of Ahi1 KO mice (Fig. 7). These data suggested that Ahi1 deficiency results in an animal model with depressive profiles.

### Ahi1 deficiency decreased depression-related neurotransmitters

Several hypotheses have been proposed for the mechanisms of major depression. The monoamine hypothesis of major depression has dominated industrial and academic research for the past five decades. It suggests a deficiency or imbalance in monoamine neurotransmitters such as serotonin, dopamine, and norepinephrine is related to depression. Serotonin deficiency have been reported in the brains of depressed suicide victims [19]. This hypothesis has also been supported by the fact that either early versions of antidepressants including tricyclics and monoamine oxidase inhibitors or recent selective serotonin reuptake inhibitors (SSRIs) have the common effect of enhancing monoamine function [20], [21]. Low levels of serotonin, dopamine or norepinephrine were also found in different depression animal models [22]. In our Ahi1 KO mice, we also confirmed low levels of serotonin and dopamine by HPLC method. At the beginning of birth, these neurotransmitters were at same level in control and Ahi1 KO mice (Fig. 2); however, after postnatal day 10, serotonin and dopamine levels were significantly increased in control brains, but not in Ahi1 KO mice (Fig. 2). Therefore, depressive behaviors were observed in 1-m-old Ahi1 KO mice (Fig. 1). Serotonin and dopamine levels decreased not only in depression-related brain regions such as hippocampus, amygdale, and hypothalamus, but also in other regions like cortex and brainstem (Fig. 2–4). On the contrary, levels of non-monoamine neurotransmitters including GABA and glutamate did not change. How Ahi1 deficiency causes low levels of serotonin and dopamine is still under investigation, but one possibility may be explained by high MAO activity in brains of Ahi1 KO mice (Fig. 6) because MAO metabolizes monoamines [16]. In addition, the ratio of 5-HIAA to serotonin was significantly increased in Ahi1 KO mice, but the ratio of HVA to dopamine was not changed in control and Ahi1 KO mice (except hypothalamus). These indicated that serotonin has higher turnover than dopamine in Ahi1 KO mice and serotonin and dopamine may have different degradation mechanisms.

From our data, most neurotransmitters in normal brains are at low levels after birth and increases with age. These neurotransmitters level reach the peak and become stable from the age of 1 m. However, Ahi1 KO may affect the regulations of neurotransmitter synthesis, transportation, or release after birth, but not in pre-natal brains; therefore, neurotransmitters such as serotonin and dopamine does not increase significantly with age in Ahi1 KO mice. Consistent with neurotransmitter results, depressive behaviors appear in 1-m-old Ahi1 KO mice and become a stable phenotype. Therefore, our findings support the monoamine hypothesis and demonstrate the basis of stable depressive phenotype of Ahi1 KO mice.

### Ahi1 KO mice are a genetic model of depression for the mechanistic study and screening therapeutic targets

A good animal depression model can provide an opportunity to understand molecular, genetic, and epigenetic factors that may lead to depression, afford a good insight of pathology of depression, and offer a tool to identify novel therapies for depression. So far, there is no ideal animal model that perfectly reproduces the symptoms of depression in human. In addition, the hallmarks of depression such as depressed mood, low self-esteem or suicidality are hardly evaluated in animal models. However, as an animal model of depression, it should contain some endophenotypes including anhedonia, behavioral despair, neuroanatomical changes, neuroendocrine disturbances, and alterations in sleep architecture as described before [23]. Therefore, The criteria for ideal animal models of depression was proposed as followings: strong phenomenological similarities and similar pathophysiology, comparable etiology, and common treatment [24]. In this study, anhedonia in Ahi1 KO mice was assessed by sucrose preference and confirmed its existence; behavioral despair was evaluated by forced swimming and tail suspension tests; our unpublished data demonstrated neuroendocrine disturbances including the increased serum glucocorticoid level. In addition, the changes of neurotransmitters including serotonin and dopamine and MAO activity supported the monoamine hypothesis. Therefore, Ahi1 KO mice are a good genetic model of depression for pathologically mechanistic study. Importantly, these mice also responded to a known antidepressant impramine that decreased immobility time and increased brain serotonin level. All these findings supported that Ahi1 KO mice are a good depression model for screening therapeutic targets.

## Conclusions

In summary, Ahi1 deficiency contributes to depression-like behaviors together with the decrease of monoamine neurotransmitters and high level of MAO activity. The characterizations of Ahi1 KO mice demonstrated that Ahi1 KO mice is a good genetic model of depression and can be used for studying the mechanism of depression and screening therapeutic targets.
