# Correction: Loss of Ahi1 Impairs Neurotransmitter Release and Causes Depressive Behaviors in Mice

**DOI:** 10.1371/journal.pone.0103802

**Published:** 2014-07-22

**Authors:** The PLOS ONE Staff

There is an error in the grant number in the Financial Disclosure (FD) statement for this article. Please refer to the correct FD statement below:

This study was supported by grants 81071095, 81120108011, and 81361128010 (to XX) from the National Natural Science Foundation of China, Suzhou science and technology development program (SYS201232), the Priority Academic Program Development of Jiangsu Higher Education Institutions of China, Jiangsu Province’s Key Provincial Talents Program (to XX), and the National Basic Research Program of China (973 Program, 2013CB945401, to MS). The funders had no role in study design, data collection and analysis, decision to publish, or preparation of the manuscript.

## References

[1] Ren L, Qian X, Zhai L, Sun M, Miao Z, et al (2014) Loss of Ahi1 Impairs Neurotransmitter Release and Causes Depressive Behaviors in Mice. PLoS ONE 9(4): e93640 doi:10.1371/journal.pone.0093640 24691070 10.1371/journal.pone.0093640 PMC3972168

